# Communities in charge of alcohol (CICA): a protocol for a stepped-wedge randomised control trial of an alcohol health champions programme

**DOI:** 10.1186/s12889-018-5410-0

**Published:** 2018-04-19

**Authors:** Penny A. Cook, Suzy C. Hargreaves, Elizabeth J. Burns, Frank de Vocht, Steve Parrott, Margaret Coffey, Suzanne Audrey, Cathy Ure, Paul Duffy, David Ottiwell, Kiran Kenth, Susan Hare, Kate Ardern

**Affiliations:** 1 School of Health Sciences, University of Salford, Manchester, UK; 2 School of Health & Society, University of Salford, Manchester, UK; 3 Population Health Sciences, Bristol Medical School, University of Bristol, Bristol, UK; 4 School of Health Sciences, University of York, York, UK; 5 Public Health England North West, Manchester, UK; 6 Greater Manchester Combined Authority, Manchester, UK; 7 Royal Society of Public Health, London, UK; 8 Fallowfield Community Guardians c/o School of Health Sciences, University of Salford, Manchester, UK; 9 Wigan Council, Manchester, Wigan UK

**Keywords:** Alcohol, Public health, Asset based community development, Brief intervention, Licensing, Dark logic, Community-based prevention

## Abstract

**Background:**

Communities In Charge of Alcohol (CICA) takes an Asset Based Community Development (ABCD) approach to reducing alcohol harm. Through a cascade training model, supported by a designated local co-ordinator, local volunteers are trained to become accredited ‘Alcohol Health Champions’ to provide brief opportunistic advice at an individual level and mobilise action on alcohol availability at a community level. The CICA programme is the first time that a devolved UK region has attempted to coordinate an approach to building health champion capacity, presenting an opportunity to investigate its implementation and impact at scale. This paper describes the protocol for a stepped wedge randomised controlled trial of an Alcohol Health Champions programme in Greater Manchester which aims to strengthen the evidence base of ABCD approaches for health improvement and reducing alcohol-related harm.

**Methods:**

A natural experiment that will examine the effect of CICA on area level alcohol-related hospital admissions, Accident and Emergency attendances, ambulance call outs, street-level crime and anti-social behaviour data. Using a stepped wedged randomised design (whereby the intervention is rolled out sequentially in a randomly assigned order), potential changes in health and criminal justice primary outcomes are analysed using mixed-effects log-rate models, differences-in-differences models and Bayesian structured time series models. An economic evaluation identifies the set-up and running costs of CICA using HM Treasury approved standardised methods and resolves cost-consequences by sector. A process evaluation explores the context, implementation and response to the intervention. Qualitative analyses utilise the Framework method to identify underlying themes.

**Discussion:**

We will investigate: whether training lay people to offer brief advice and take action on licensing decisions has an impact on alcohol-related harm in local areas; the cost-consequences for health and criminal justice sectors, and; mechanisms that influence intervention outcomes. As well as providing evidence for the effectiveness of this intervention to reduce the harm from alcohol, this evaluation will contribute to broader understanding of asset based approaches to improve public health.

**Trial registration:**

ISRCTN 81942890, date of registration 12/09/2017.

## Background

Reducing alcohol related harm continues to be a global public health priority, with 5.9% of all global deaths and 5.1% of all disease and injuries being attributable to alcohol [1]. Excessive alcohol consumption harms an individual’s health and social relationships [2]. It also harms society more generally, as urban areas can become less pleasant and less safe to visit [3] and crime may increase [4]. Moreover, the consumption of alcohol contributes significantly to health inequalities. Those living in deprived areas drink the same average quantity of alcohol as those from more advantaged groups. However, a so-called ‘alcohol harm paradox’ exists whereby, for a given level of alcohol consumption, alcohol harm is higher amongst those living in more deprived areas [5]. Possible reasons for this include patterning of consumption (e.g. consuming the same quantity on fewer occasions [6]) and combinations of other health risks (e.g. smoking, obesity) in individuals living in deprived areas [7]. Interventions that are effective at reducing alcohol harm have been shown to operate at the individual level (i.e. brief advice about drinking [8]), the community level (e.g. licensing policies [9, 10] and measures that control access to alcohol [8]), and national level (e.g. alcohol pricing policy [2]). This protocol describes an evaluation of an intervention, ‘Communities In Charge of Alcohol’ (CICA), which aims to target alcohol harm at two levels; by influencing individuals (through brief intervention) and communities (through reducing the availability of alcohol).

Brief interventions and brief advice have been shown in systematic reviews and meta-analyses to be effective in a variety of settings including emergency departments [11] and primary care [8]. There is relatively little evidence about training lay persons for this role, although pilot work with ex-offenders giving advice to offenders in community settings seems promising [12]. Accessibility of alcohol is a key determinant of harm [5, 13, 14]. Internationally, systematic review evidence shows that high alcohol outlet density is linked to higher levels of crime and poor health [15]. A systematic review (rated high quality [16]) found that higher outlet density and greater exposure to advertising tends to be associated with higher levels of alcohol use [17]. Interventions that change the alcohol environment thus have the strongest evidence for effectiveness [2, 16, 18].

In England, local authorities can address public health through licensing policies. However, because ‘public health’ is not currently one of the licensing objectives, the extent to which this is carried out varies across the country [9]. Local people have the ability to influence the availability of alcohol via the licensing process, but do not tend to do so, due to low awareness and lack of confidence that local views will be valued [19]. Recent longitudinal, area-level analysis of UK datasets shows that, at borough level (i.e. lower tier local authorities in England) both alcohol-related hospital admissions [9] and crime [10] have reduced faster in areas where more restrictive licensing policies are in place. Using small area level data (lower super output areas [LSOA^1^] with a mean population of 1500 persons), alcohol outlet density in Wales is similarly associated with alcohol-related hospital admissions and crime data [5]. The possibility of utilising community advocacy to reduce the availability of alcohol has not been explored but could represent an untapped resource. This aligns to the UK Government’s attempts to make it easier for residents and other local agencies interested in licensing to take action [20].

The CICA programme takes an Asset Based Community Development (ABCD) approach, where a health asset is any factor which enhances the ability to create or sustain health and wellbeing [21]. This is in line with the National Institute for Health and Care Excellence (NICE) guidance on behaviour change, which advocates building on existing community resources and skills [22]. The principles of the approach are to: allow time for communities to realise and acknowledge their individual and collective assets and to rebuild their confidence and networks; enable local people to take the lead, and; build trust with communities by demonstrating that involvement leads to change [23]. The approach seeks to build community networks, which are health promoting [24]. The programme aims to enable local volunteers working with the community to identify alcohol harm in their community, and facilitate them to intervene to reduce these harms.

## Methods/design

The overarching aim of this research is to evaluate the effectiveness of a community Alcohol Health Champions programme (Communities in Charge of Alcohol: CICA) to reduce alcohol-related harm. Secondary aims include determining the cost consequences of CICA and exploring the context, barriers and facilitators to its implementation. As a complex community intervention, it is not amenable to conventional randomisation (as recognised in the complex interventions guidance [25]) and will be evaluated as a natural experiment [26]. This fits on the ‘continuum of evaluation’ [27], which recognises the need for multiple methods/variants on experimental design [28].

Through CICA, individuals embedded within areas identified as having high levels of alcohol-related harm are recruited by local co-ordinators to take part in a 2 day ‘Alcohol Health Champion’ (AHC) course. As a community asset connected to the area either through their residency or their work role, an Alcohol Health Champion’s existing strengths, motivations and skills are strengthened to enable them to (i) give alcohol-related brief advice to others and (ii) tackle the availability of alcohol in the local environment through the licensing process. Using a cascade training model, an accredited and standardised training course is delivered initially by the Royal Society for Public Health (RSPH) and then cascaded by the Alcohol Health Champion community with the support of professionals. This community hub approach means that AHCs have an infrastructure of support provided by local co-ordinators and local licensing officers. The AHC role description specifies that champions should only do whatever they are comfortable with and that the programme does not dictate where, when or how much activity should take place. This is largely based on the existing health champions model which utilises lay health workers from a variety of backgrounds (e.g. voluntary sector, housing, local residents) to work in a voluntary capacity to offer brief advice and brief interventions alongside their other daily activities [29]. However, for the first time, through CICA, champions will be trained to focus on alcohol and will receive additional knowledge and skills to enable them to get involved in local licensing decisions.

**Fig. 1:**
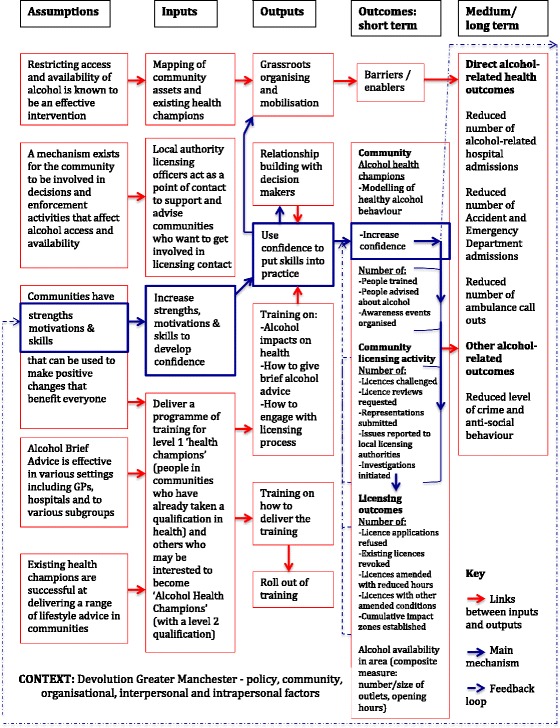
CICA Evaluation Logic Model

A logic model was created as part of the planning of this evaluation (Fig. 1). This shows the intervention’s mechanisms of action and the interplay between its core components. At the heart of the CICA programme, based on ABCD principles, is the assumption that individuals and communities have strengths, motivations and skills that benefit everyone. Further, there is an assumption that the AHC training programme and infrastructure of support can help build the strengths, motivations and skills of these individuals to develop confidence to put their skills into practice. As communities take action by offering brief advice or getting involved in licensing decisions, a feedback loop illustrates how such success positively reinforces the strengths, motivations, and skills of the community. Influencing access and availability to alcohol and building a groundswell of brief advice about alcohol should, as a result, directly impact on alcohol related outcomes.

**Fig. 2:**
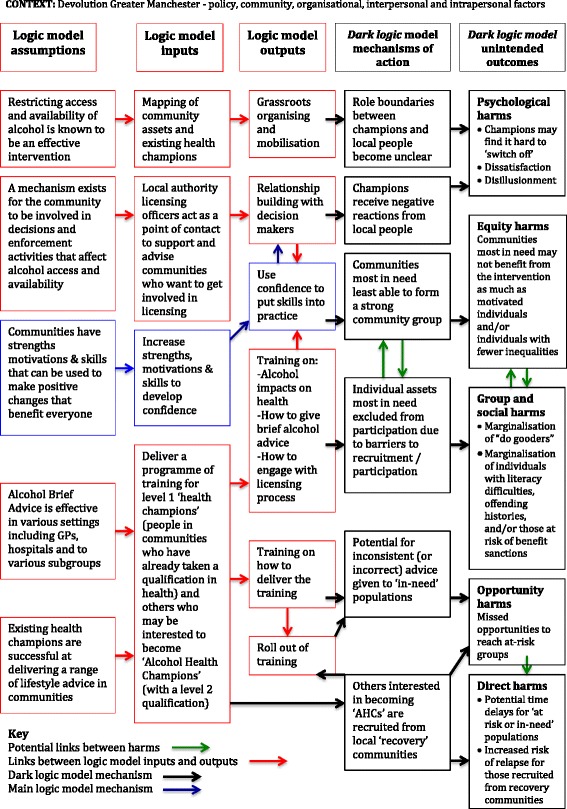
CICA “Dark” Unintended Consequences Logic Model

Additionally, we also considered the possible unintended consequences of the intervention (see Fig. 2). Few public health interventions and evaluations explicitly look at unintended harms and, although logic models considering positive consequences and outcomes are common, the consideration of the potential negative outcomes (and their mechanisms of action), or a ‘dark’ logic model, are less common [30]. According to Bonell et al. [31], not only is it important to produce a dark logic model ahead of the evaluation/intervention, or during it, but also the framework could be useful to evaluate the project retrospectively to see how it might have been strengthened. To develop the dark logic model we first created a matrix to hypothesise *a priori* the potential unintended consequences of CICA using a simple framework, adapted from Lorenc and Oliver’s five categories of harm (see Table 1) [30]. Each potential harm was reflected upon using one of three approaches recommended by Bonell et al. [31]: how agencies and structures may interact in unintended ways; comparative understanding across similar interventions; and consultation with individuals/groups with insights into local contexts and how interventions might operate within them. Evidence that supports or refutes the hypothesised unintended consequences of the intervention will benefit future design and minimise the risk of future harm [31].

**Table 1:** CICA Matrix of potential unintended consequences

Potential unintended consequences	How agencies and structures may interact in unintended ways	Comparative understanding across similar interventions	Consultation with individuals/groups with insights into local contexts and how interventions might operate within them (CICA Project Advisory Group)
Direct harms	None identified	Lack of depth of knowledge by lay health advisors could result in time delays or inconsistent advice for ‘in-need’ populations [48]	Concerns that volunteers recruited from recovering communities could be at increased risk of relapse of alcohol, drug or mental health problems
Psychological harms	None identified	Volunteers embedded within communities find it hard to ‘switch off’ [48]	Intervening in licensing could lead to negative reactions from local retailers
		Dissatisfaction and disillusionment of volunteers [49]	
Equity harms	Communities most in need are probably the least able to form a strong community group [50, 51]	Motivated individuals becoming health champions are likely to benefit from being a champion more so than those less motivated (who need the potential positive benefits more) [48]	Individual assets within communities excluded from participation due to barriers to recruitment/participation e.g. literacy, criminal record checks, worry about impact on benefits
Group and social harms	‘Communities’ chosen to be in charge of alcohol set by experts (normative needs) vs. self-identified communities (felt needs) [49]	Becoming a community champion could result in lack of acceptance by own community resulting in marginalising “do gooders” [48]	None identified
		Current recovery dominated culture within alcohol service provision in UK influences the selection of volunteers from ‘recovery’ communities [52]	
Opportunity cost harms	Commissioners may miss opportunities to invest in alternative public health interventions [53]	Missed opportunities to identify “at-risk” populations [54] due to stereotyping those ‘in need’ as only the most severe drinking patterns [55]	None identified

### Setting

All Greater Manchester (GM) boroughs within the study setting have higher than England averages for alcohol-related mortality, ranging from 46.7 in Trafford to 71.9 per 100,000 in Manchester [32]. GM is heterogeneous in terms of its application of licensing policy: in a recent national study, only one local authority was classified as having high licensing policy intensity (two local authorities were medium, five low and two passive in terms making use of cumulative impact areas and/or declining licences [9]).

CICA will be rolled out sequentially across specifically targeted areas in all 10 local authorities, with the sequence randomly assigned by the research team, so that it will eventually (within the timespan of a year) be delivered in all areas. The intervention areas themselves within each local authority will be formed around pre-existing communities in LSOA locations. For data analysis purposes, the chosen LSOAs that represent each community combine to make the ‘intervention area’ unit of analysis. For example, the smallest intervention area encompasses one LSOA (mid-year population estimate of 1648) and the largest contains three LSOAs (mid-year population estimate combined of 5586).

### Characteristics of study population

In order to ensure there is consistency for the evaluation, each local authority used the following guiding principles as inclusion criteria for selecting an intervention area dependent on local available data:

- An area of high alcohol-related harm (defined as high within the local authority, rather than in comparison to regional or national average rates)
- Alcohol harm considered in terms of a combination of indicators:

- Alcohol-related crime^2^ and anti-social behaviour (ASB)^3^
- Alcohol-related hospital admissions
- Weekend evening Accident and Emergency (A&E) attendances
- Users of local treatment services
- Hospital recording of location of violent incidents (if available)
- Density of licensed premises in the area or adjoining areas (if available)

The study population of AHCs will include adults aged 18 years and over, who are already embedded in the intervention area either through their residency or their work role (i.e. they must spend the majority of their time in the intervention area).

### Outcome evaluation

The effectiveness of CICA will be measured through the following data and objectives:

- Routinely collected quantitative data to determine the effect on key health performance indicators (narrow indicator of alcohol-related hospital admissions,^4^ A&E attendances and ambulance call-outs);
- Routinely collected street-level crime data to determine the effect on key crime indicators;
- Routinely collected anti-social behaviour data to determine the effect on key ASB indicators.

The aims and objectives of the outcome evaluation will be reached through outcome analysis at the level of the small intervention area (the equivalent of one to three LSOAs) comparing areas where CICA has not yet been introduced. The outcome analysis involves two distinct approaches:

- ***‘Internal’ evaluation***: Trends in area-time intervention areas will be compared before and after the intervention using a stepped-wedge randomised trial design [33]. Sensitivity analyses using different lagging periods (6–24 months) between introduction of the intervention and expected effects will allow for a delayed effect on output measures. If differences in the slopes of the longitudinal models are observed, the population impact will be estimated from deviation of the post-intervention slope compared to the pre-intervention slope.
- ***‘External’ evaluation***: Secondly, the impact of the intervention will be assessed using two complementary methods: (i) matching intervention and control areas inside the GM area by area-level deprivation, population size, age distribution and baseline alcohol-related burden by calculating propensity scores [34]. Temporal trends in each of the outcomes will be plotted graphically and analysed using hierarchical growth models (similar to de Vocht et al. [9, 10]); and (ii) using time series data from GM LSOAs to construct weighted ‘synthetic control time series’ [35] that mimic the ‘intervention area’ as closely as possible prior to the introduction of CICA. Modelled and measured post-introduction time series will then be compared directly to quantitatively estimate the impact of CICA. Both methodologies (i and ii) are complementary, and while the latter approach has the distinct advantage that it provides a direct comparison to the counterfactual time-series, it can be considered as less insightful than the former method because it does not compare actual areas on the ground.

The external evaluation may be affected by ‘spill-over’. In other words, if, as a result of the introduction of CICA, it becomes more difficult for new premises to obtain a premises licence in the specific intervention areas, these may decide to establish themselves in neighbouring areas (close to the border). Conversely, AHCs could get involved in licensing decisions in these neighbouring areas directly. These are known ‘spill-over’ issues [36], but difficult to tackle, and therefore directly neighbouring LSOAs will not be incorporated into control areas. Instead, only LSOAs that are further away are combined and matched, as outlined above, using propensity score matching or incorporated in the synthetic control time series.

### Statistical analysis and power calculation

Statistical power calculations were based on the methodology for stepped-wedge randomised trials outlined in Hussey and Hughes [33]. These were calculated specifically for the primary outcome ‘alcohol-related hospital admission rates (narrow)’ obtained from the Local Alcohol Profiles for England (LAPE)^4^ extracted for all 10 GM local authorities. Statistical power analyses were conducted at local authority level rather than at the level of the intervention area (the equivalent of one to three LSOAs) because the exact areas and comparisons had not been determined, but this aggregated level provided indications within the stepped wedge context. The mean standardised alcohol-related hospital admission rate in these local authorities for the year 2014 was 207 (per 100,000 people) with a maximum temporal standard deviation per site of 17.2 (range within sites 5–17) and a coefficient of variation across sites of 4.35. With 10 areas and 12 month follow-up (i.e. when all areas have received the intervention and a minimum of 1-month post-intervention follow-up), and a statistical significance level of 5% and statistical power of 90%, the proposed study will be able to detect a 10% average difference in rates compared to baseline. For an intervention to be effective and cost-effective a minimal reduction in key indicators of 10% seems reasonable. Statistical analyses will use standard mixed-effects models with an indicator of when the intervention was introduced in each area and a time component to account for the repeated measures nature of the data.

With respect to the comparison with propensity matched controls and synthetic controls in which time trends will be compared within the larger area of between different areas, no additional clustering occurs. Assuming a standard comparison of independent means, 1-sided test, and significance level of 5%, changes in alcohol-related hospital admission rate in the intervention LSOA, relative to the selected comparison area, yield an 84% statistical power to detect a similar 10% decrease. Trends in matched areas are evaluated using mixed-effects log-rate models as previously used at lower-tier local authority area level for alcohol-related hospital admissions [9] and alcohol-related crime rates [10] in England.

To create the synthetic controls Bayesian structural time series methods will be used [37, 38]. These ‘synthetic areas’ will be based on weighted averages of other GM local areas, where the weights are chosen so the synthetic GM area most closely resembles the actual GM area before the intervention started [39]. Trends between the measured and the modelled, counterfactual, outcomes in the synthetic controls (generated using Bayesian structural time series) will be interpreted as the intervention effect.

In these statistical power calculations we have not taken into account any potential ‘spill-over’ effects, such as described for a community action programme in Sweden [40], and which implies that the above may be an underestimation of the true statistical power (or, conversely, of the minimal detectable effect). It is unclear how these should be modelled, and therefore, as outlined above, no LSOAs immediately adjacent will be matched.

### Economic evaluation

The economic evaluation aims to conduct a cost consequences analysis of the CICA programme. It will do this through:

- Identifying set-up and running costs using a standardised costing exercise (examination of commissioning documents and contracts);
- Resolving costs by sector (health, ambulance and police) before, during and after the CICA set up;
- Quantifying benefits due to reduced hospital admissions, ambulance call outs, emergency department use, crime and anti-social behaviour.

The costs of training, delivery and support elements of the intervention will be estimated. The economic evaluation will build on the outcome evaluation by attributing costs to the health performance indicators collected on primary outcomes. UK Treasury approved methods published by New Economy will underpin the cost-consequences analysis (CCA) and unit costs are based on the New Economy Unit Cost Database [41].

A standard costing exercise will use documents and contracts to identify resources and costs required to deliver the CICA intervention in each local authority. Standardised methods allow comparability of costs. The economic evaluation follows a cost-consequences analysis which is an approach that is favoured when costs and outcomes fall on a range of budget-holders and government agencies enabling cost and consequence domains to be presented in a disaggregated form [42, 43]. This will enable decision makers to assess results using different relevant perspectives.

The key cost categories identified include the set up cost for the intervention area, comprising staff costs, consumables and overheads (premises). In terms of consequences, this comprises an analysis of health benefits, changes in health care resource utilisation as a consequence of alcohol use (A&E attendances, hospital inpatient stays, ambulance service costs), and changes in contacts with the criminal justice system.

The outcome analysis uses mixed-effects log-rate models and differences-in-differences models to evaluate changes relative to propensity-score-matched controls and will use Bayesian structural time series to model the synthetic control areas; both to assess and compare potential changes in the health care and criminal justice resources before and after CICA interventions. The economic component of the study will follow the same statistical methods used in the outcome analysis and applies unit costs to the resource indicators to derive costs for each domain. These costs will be presented in a CCA framework, disaggregated in terms of costs to individual stakeholders and different cost domains.

### Process evaluation

The design of the process evaluation was informed by MRC guidance for conducting process evaluation of complex public health interventions [28]. The aim is to explore the factors that enable or hinder the implementation of the intervention. This includes establishing, operationalising and sustaining the CICA intervention. The objectives of the process evaluation are as follows:

- Exploring the policy context and variation in licensing practice, including any impact of devolution in Greater Manchester;
- Explore barriers/facilitators at key stages of the intervention (recruitment of AHCs to initial training and cascade training, delivery of initial training and cascade training, using skills beyond the training in AHC activity; retention of AHCs);
- Explore response to AHC training, modelling of health behaviours, perceptions of community cohesion and development;
- Determine the numbers of trainees, brief interventions applied and community awareness events organised/participated in;
- Examine and quantify the amount and success of community involvement in licensing issues;
- Determine whether there is a change in composite measures of alcohol availability.

**Fig. 3:**
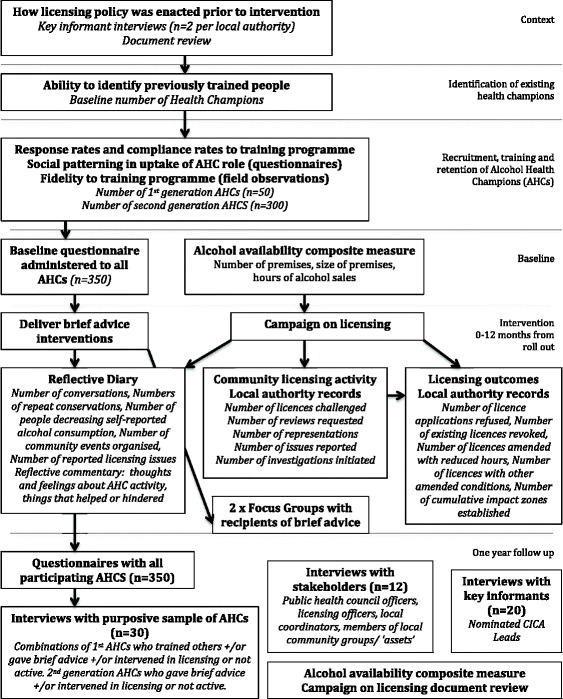
Charted summary of process evaluation methods

The aims and objectives of the process evaluation will be achieved using mixed methods to examine the context, acceptability, facilitators and barriers to the intervention (see Fig. 3). Appropriate analysis techniques have been selected for each method. Documents will be analysed using content analysis, and quantitative data from documents (e.g. on numbers of licence applications, reflective diaries completed by AHCs) will be extracted and described. Interviews and focus groups will be digitally recorded, fully transcribed and anonymised. Analysis will utilise the Framework method [44]: textual data will be ‘charted’ in themes relating to key research questions and scrutinised for differences and similarities within themes, keeping in mind the context in which these arise. We will ‘sense check’ our emerging qualitative findings with stakeholders, who will help us with the interpretation of our themes.

Questionnaire data with AHCs (at baseline and 1 year follow-up) and data on numbers of licences challenged will be analysed using descriptive statistics (SPSS v23). Data will be analysed to construct the most robust and plausible explanation of observed outcomes. The logic model, programme theory and ‘dark logic’ model may be modified in the light of study findings.

Reflective diaries will be completed by AHCs who consent, although the detail will be as much or as little as the AHC is willing to provide. The benefits of completing a reflective diary include supporting the AHC to learn from their experiences by looking back on facilitators and barriers to carrying out their roles beyond the initial CICA training [45] and for the evaluation team in understanding the experiences of the AHCs over the intervention period. Reflective diaries have been used successfully in other public health interventions, for example, helping peer supporters in a school-based stop smoking intervention: however, it was acknowledged that the information provided was not a full and accurate indication of the peer-supporters’ conversations and interactions [46]. The research team evaluating the CICA project recognise that reflective diaries cannot be used for monitoring the actions of the AHCs and rather aim to give a flavour of the activities and the reflections of “being an Alcohol Health Champion” over the intervention period. This will then be followed up with a sample of more in-depth interviews where experiences can be explored in greater depth, whether or not these experiences are recorded in the diaries.

## Discussion

The ABCD approach is currently being promoted widely (e.g. in new NICE guidance [29]) and is attractive in terms of current fiscal challenges and cuts to services, but there is relatively little evidence for its effectiveness [47]. Therefore, we anticipate the findings will be widely relevant across a range of topics, not just interventions to reduce alcohol harm. The results will be of interest to policy makers, commissioners and public health practitioners responsible for reducing alcohol harm at both a population and individual level. Evidence on the context within which community participation programmes for health improvement are implemented will help increase knowledge about their mechanisms of action and potential inaction. Evidence on the cost-consequences of CICA will quantify its set up and running costs and whether there are any benefits to reduced hospital admissions, ambulance call outs, emergency department use, and crime.

As a pragmatic study evaluating a community response to a training and support programme, there is likely to be variation in the degree to which the intervention is applied, and the exact nature of the activities in each area. However, the scale of the intervention and the methodology, which includes an element of randomisation and an evaluation of promising new methods for analysing natural experiments, alongside the health economic analysis, are significant strengths of this study. The process evaluation and analysis of the logic and dark logic models will help us to evaluate the specific context, the particular actions or responses involving a given set of actors, and how these are responsible for generating given outcomes. These strengths should enable a comprehensive assessment of the CICA programme.

## Abbreviations

A&E: Accident and emergency
ABCD: Asset based community development
AHC: Alcohol health champion
ASB: Anti-social behaviour
CCA: Cost consequences analysis
CICA: Communities in charge of alcohol
GM: Greater Manchester
GMCA: Greater Manchester combined authority
HES: Hospital episode statistics
LAPE: Local alcohol profiles for England
LSOA: Lower super output area
NICE: National Institute of Health and Care Excellence
NIHR: National Institute for Health Research

## Footnotes

1 Lower super output area (LSOA) is a unit of measurement generated for small area statistics; they are consistent in size of population, reflect the characteristics of census data where possible and are stable boundaries https://www.ons.gov.uk/methodology/geography/ukgeographies/censusgeography#output-area-oa, accessed November 2017. http://www.datadictionary.nhs.uk/data_dictionary/nhs_business_definitions/l/lower_layer_super_output_area_de.asp?shownav=1, accessed November 2017.

2 Crime data relates to those events where a crime (in law) has occurred, and where this is included within the Home Office Counting Rules for Recorded Crime (i.e. a ‘notifiable’ crime as defined within the National Crime Recording Standard), Home Office (2017) Counting Rules for Recorded Crime, https://www.gov.uk/government/publications/counting-rules-for-recorded-crime, accessed January 2018.

3 Anti-social behaviour data relates to those events reported to the police where a crime has not occurred, but where the police receive a call for service and the recorded incident is classified by the call handler as involving anti-social behaviour, in accordance with national guidance, Home Office (2011) National Standard for Incident Recording (NSIR), https://www.gov.uk/government/publications/the-national-standard-for-incident-recording-nsir-counting-rules, accessed January 2018

4 The narrow indicator of alcohol-related hospital admissions measures the number of individuals admitted to hospital due to a primary diagnosis with an alcohol-attributable code or any secondary diagnosis with an external alcohol-attributable code, Public Health England (2015) Local Alcohol Profiles for England user guide 2015, http://webarchive.nationalarchives.gov.uk/20171107173500/http://www.lape.org.uk/downloads/LAPE%20User%20Guide_Final.pdf, accessed December 2017
